# Parental Knowledge and Acceptance of Different Treatment Options for Primary Teeth Provided by Dental Practitioners

**DOI:** 10.3389/fpubh.2019.00322

**Published:** 2019-11-07

**Authors:** Ola B. Al-Batayneh, Hanan O. Al-Khateeb, Waiel M. Ibrahim, Yousef S. Khader

**Affiliations:** ^1^Pediatric Dentistry Division, Preventive Dentistry Department, Faculty of Dentistry, Jordan University of Science and Technology, Irbid, Jordan; ^2^Department of Public Health and Epidemiology, Faculty of Medicine, Jordan University of Science and Technology, Irbid, Jordan

**Keywords:** parental knowledge and acceptance, treatment options, primary teeth, caries prevention, DMFT/dmft, children

## Abstract

**Background:** Parents have an influence on dental treatment options for young children regarding type of care provided. The aim of this study was to assess parents' knowledge and acceptance of different treatment options for primary teeth provided by dental practitioners for their children.

**Materials and Methods:** In this descriptive, cross-sectional study, caregiver/child dyads (*n* = 476) were recruited from patients at Pediatric Dental Clinics, Jordan University of Science and Technology. The data collection questionnaire to parents included: 1-demographic data 2-parental knowledge and practices regarding child's oral hygiene, caries and caries prevention 3-parental knowledge and acceptance of different treatment options for primary teeth including two given clinical scenarios (ICDAS-5 molar requiring intra-coronal restoration, ICDAS-6 molar requiring pulp therapy and stainless steel crown) with pictures before and after treatment. Afterwards, the child underwent a dental examination to record dmft/DMFT, gingival and plaque indices. Data was analyzed using SPSS, significance was set at *P* ≤ 0.05.

**Results:** Children's ages were 2–12 years (mean/SD 6.97 ± 2.5); with 255/53.6% males, 221/46.4% females. There were (166) children 2–5 years in primary dentition; (108/166) 65% had ECC, and (*n* = 62/166) 37.4% had S-ECC, and (310) 6–12 years in mixed dentition; (278/310) 89.7% had caries. Scaling and extraction were the highest known and accepted treatments for primary teeth by parents (35.5 and 30.1%, respectively), while nitrous oxide/oxygen sedation was the least (3.6%). Parental educational level was significant for composite restorations, fluoride gel application and pulp therapy (*P* = 0.03, 0.02, and 0.03, respectively) and age above 40 for amalgam restorations (*P* = 0.04). In both scenarios, most parents preferred to leave any care decision in hands of the dentists with no effect of educational level (*P* > 0.05). There were 81.5% parents who reported that their children's dental status was good, however, 78.4%/42.8% children had an average dmft/DMFT score 5.34/2.32 and mean PI/GI scores 0.88 ± 0.20/0.17 ± 0.23.

**Conclusion:** Parental knowledge and acceptance about dental treatment options for primary dentition was generally low. Parental education and age had an impact on parental knowledge and practices regarding child's oral hygiene, caries and caries prevention, and some treatment options. There was an overrated parental opinion of their child's teeth status despite the high dmft/DMFT and PI.

## Introduction

Oral health has an important role in the general well-being of individuals. The adoption of good oral health habits in childhood often takes place with parents, especially mothers (1, 2) and is affected by parental dental knowledge, attitudes, cultural beliefs and awareness about infant diet and feeding practices, oral hygiene habits, preventive regular dental visits, care of primary teeth and concern for oral health. It has been found that the more positive the parent's attitude is toward dentistry, then the better will be the dental health status of their children (3). Therefore, interventions targeting parental oral health beliefs and practices may be beneficial in the prevention of oral health problems.

Dental treatment of young children is usually provided only after explanation and consent with parents (4). There are a few studies on parental acceptance of dental treatment at the dental office for their children; some were limited to parental acceptance of dental treatment under general anesthesia or nitrous oxide, which was reported to be less than 10% for both (5); and acceptable in one third of parents in another study that evaluated acceptance of dental treatment under general anesthesia (6). In other studies, two scenarios were given to the parents to assess the acceptance of pulp therapy and restorative treatment, they found that most of the parents relied on the dentist to choose the treatment (4, 6, 7), one-third refused to do any treatment for asymptomatic teeth (4, 6), and 9–37% preferred tooth extraction for symptomatic teeth (6, 8).

Unfortunately, there is little information in the literature to show if parental age and education affects their preferences for the dental care of their children (6). The relationship between parental treatment preferences and factors such as their knowledge about oral hygiene, caries and caries prevention hasn't been fully investigated. Therefore, the aims of this study were:

1. To assess parents' knowledge and acceptance of different treatment options for primary teeth provided by dental practitioners for their children.
2. To assess parental knowledge and practices regarding the child's oral hygiene, caries and caries prevention.

## Materials and Methods

### Ethical Approval

Ethical approval was gained from the IRB, JUST (Institutional Review Board, Jordan University of Science and Technology), grant # 175/2014. In addition, written informed consent was obtained from all parents/caregivers after explanation of study objectives.

### Study Design, Setting, and Subjects

This was a descriptive, cross-sectional study with a convenient sample of families that lived in North Jordan. The sample consisted of 476 subjects who were recruited from a group of parents accompanying their children to Pediatric Dental Clinics for free dental treatment at the Faculty of Dentistry, Jordan University of Science and Technology Irbid, Jordan between April, and December, 2014, respectively. Inclusion criteria for participation were: families of a healthy child, aged 2–12 years, with primary or mixed dentition, selected randomly among the family's children where the oldest or the youngest child was chosen sequentially in the consenting family.

### Data Collection

The data were gathered by means of a self-reported questionnaire given for the primary caregiver (father or mother). A self-designed questionnaire in the local language (Arabic) was used. An investigator was responsible to choose a child randomly from each family after explaining the purpose of the study to the parent and giving instructions to completely fill the questionnaire specifically about the chosen child. Random choice of the child per family was based on choosing the only child, or choosing the eldest and youngest consecutively among each family with more than one child. Before data collection, the questionnaire was piloted twice, over a week interval, on 20 patients to ensure reproducibility, consistency and clarity; there was 95% agreement between the two times.

Two trained investigators (dentists), whose examination technique was calibrated, with (97.6%) agreement, were responsible for the child examination. Inter-examiner and intra-examiner reproducibility was measured by re-examination of 20 children participating in the study with a 1-week interval between both examinations. The *k*-value of intra-examiner reliability was calculated to be (0.97). After that, the parent was met to explain the oral health of the examined child and to clarify findings of the examination.

#### The Questionnaire and Clinical Scenarios

A questionnaire was used to collect the data in the form of 30 close-ended multiple-choice questions. The questions were split into three sections.

**Figure 1:**
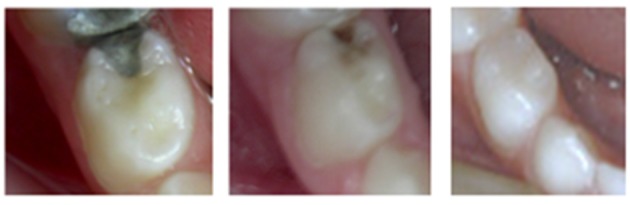
Restorative treatment ICDAS 5- “If your child has a carious asymptomatic primary tooth that needed to be restored, will you accept dental treatment or not? And if you accept the treatment, will you leave the decision of the material to be used to the dentist or will you choose between amalgam and composite?” Results: 60.3% of participants preferred to leave any care decision in the hands of the dentists, and there was no difference between university educated and secondary school level educated parents (*P* = 0.09). A few parents (8.4%) did not want any treatment to be provided since the tooth was primary and symptom-free.

**Figure 2:**
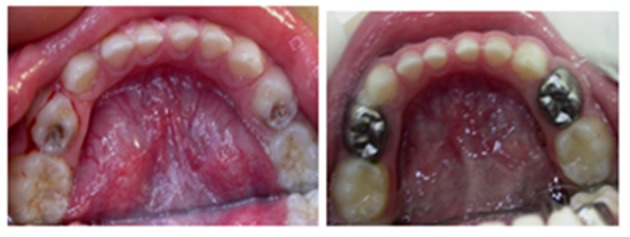
Restorative treatment ICDAS 6- Pulp therapy and Stainless steel crown-"If your child has a carious primary tooth which is causing toothache and needs pulp therapy and a stainless steel crown, will you accept dental treatment or will you choose to extract the tooth or leave it as it is? Results: 72.9% of the parents accepted the treatment option given by the dentist but 16.8% did not want any treatment. Around 11.3% of parents preferred the tooth to be extracted. Parental educational level did not play a significant role in their preferences for treatment (*P* = 0.58). Comparisons in parental choices for both clinical scenarios were not significant between children age groups (<6, 6–12) or gender.

Section 1: demographic data
Section 2: parental knowledge and practices regarding the child's oral hygiene, caries and caries prevention
*Section 3: parental knowledge and acceptance of different treatment options for primary teeth and parental opinion toward two given clinical scenarios* (Figures 1, 2).

#### Examination of the Child

From each family, the randomly selected child was fully examined intra-orally using a sterile dental front mirror and a periodontal probe (Michigan O probe with William's coding) to record dental caries, modified O'leary gingival and plaque indices (0 = absence, 1 = presence on sextant primary teeth). Dental caries was recorded at baseline using WHO Oral Health Survey Basic Methods 1997 criteria; dmft/DMFT index revealed decayed (d/D), missing/extracted (m/M) and filled (f/F) teeth (t/T) in deciduous/permanent teeth (9). The modified index was used in previous studies (10) and was based on the O'Leary index (11) and The Plaque Assessment Scoring System (12) was used in obtaining the plaque index (PI) score. Measurements were obtained by recording plaque deposits on all surfaces (buccal, lingual, mesial and distal) of the following teeth: 16/55, 21/61, 24/either 64 or 63, 46/85, 41/81, 44/84. If plaque was visible on the probe, the surface was counted as positive for plaque accumulation and given a score of 1 for plaque presence at that surface otherwise a score of 0 for no plaque. There were 24 possible plaque surfaces per patient. Gingival index (GI) scores were obtained for the teeth using the same method in scoring PI. Gingivitis was considered present if there was bleeding on probing clinically. The plaque/ gingival index for the child is the percentage of surfaces positive for plaque/gingivitis. For both indices, the score of the patient would range between 0 and 1. Based on a previous study, patients were considered not to have plaque or gingivitis if the score was between 0 and 0.13; if the score of patients was from 0.17 to 1.0 they were considered to have plaque, calculus or gingivitis (10). Subjects were rewarded for cooperative behavior by gifts.

### Sample Size Calculation

The sample size was calculated assuming that 50% of parents had proper knowledge and practices regarding child's oral hygiene, caries and caries prevention. The assumption of 50% was used to yield the largest sample size. At a power of 80% and level of significance of 0.05, the sample needed was estimated at 385 for estimating the expected proportion with 5% absolute precision.

### Statistical Analysis

Data entry and statistical analysis were done using Statistical Package for the Social Sciences (SPSS version 25.0) for windows (SPSS Inc., Chicago, USA). Means and standard deviations for the variables were calculated. The Chi-square test was used to compare percentages. Multivariate analysis using binary logistic and multinomial regression was conducted to test the differences in parental knowledge and practices according to parental age and education. A *P* ≤ *0.05* was considered statistically significant.

## Results

The response rate was 91.5% (*n* = 526/575). Of the 526 parents/children who participated in this study, 50 forms were excluded due to incomplete data or examination. The remaining 476 forms were analyzed. Parental demographic data are shown in Table 1. The children's ages ranged between 2 and 12 years with a mean (SD) age of 6.97 (2.5) years. Of the total examined children, 255 were males (53.6%) and 221 were females (46.4%). For the sake of analysis, the children were divided into two groups; ages <6, years (*n* = 166 children, with primary dentition), and ages 6–12 years (*n* = 310, in the mixed dentition).

**Table 1:** Parental demographic data according to children's age (years), total sample *n* = 476 (<6 years old = 166, 6–12 years old = 310), % are based on the total sample size.

**Variable**	**Father N (%)**		**Mother N (%)**	
	**Children <6**	**Children 6–12**	**Children <6**	**Children 6–12**
**Age (years)**				
20–40	54 (11.3)	82 (17.2)	93 (19.5)	123 (25.8)
Total	136 (28.6)		216 (45.4)	
More than 40	14 (2.9)	74 (15.5)	5 (1.1)	31 (6.5)
Total	88 (18.5)		36 (7.6)	
**Education**				
Secondary school	22 (4.6)	41(8.6)	31 (6.5)	68 (14.3)
Total	63 (13.2)		99 (20.8)	
University	46 (0.97)	115 (24.2)	67 (14.1)	86 (18.1)
Total	161 (33.8)		153 (32.1)	
**Total** **=** **476**	**224**		**252**	

Parental knowledge and practices toward their children's oral hygiene at home is shown in Table 2. Comparing between children age groups (<6, 6–12 years), differences existed in choosing the age appropriate toothpaste (x^2^ = 11.1; *p* < 0.001), correct tooth brushing technique (x^2^ = 78.5, *p* ≤ 0.001), caregiver supervision during tooth brushing (x^2^ = 8.1, *p* = 0.004), and additional teeth cleaning methods (x^2^ = 9.3, *p* = 0.002). In the multivariate analysis, parents aged 20–40 years and those with university education had significantly better knowledge and practices toward children's oral hygiene at home with regards to having a toothbrush, brushing frequency and supervision during brushing.

**Table 2:** Parental knowledge and practices toward their children's oral hygiene at home.

**Variable**	** <6 years old (*****N*** **=** **166)**		**6–12 years old (*****N*** **=** **310)**		**Total (*****N*** **=** **476)**		**Chi-square value**	***P*-value**
	**n**	**%**	**n**	**%**	**N**	**%**		
**Child has a tooth brush**	149	89.8	294	94.8	443	93.1	3.46	0.063
**Tooth brush bristles**							0.06	0.812
Soft	112	67.5	224	72.3	336	70.6		
Hard	37	22.3	70	22.6	107	22.5		
**Type of fluoridated tooth paste (TP) used per age group**							
Adult's TP	57	34.3	174	56.1	231	48.5	11.10	<0.001
Children's TP	109	65.7	174	56.1	283	59.5		
**Brushing frequency (daily)**							2.66	0.102
Once- Twice	119	71.7	243	78.4	362	76.1		
Never	47	28.3	67	21.6	114	23.9		
**Parent knowledge of correct tooth brushing technique**	126	75.9	310	100.0	436	91.6	78.50	<0.001
**Caregiver supervision during tooth brushing**	124	74.7	190	61.3	314	66.0	8.07	0.004
**Additional teeth cleaning methods**	22	13.3	80	25.8	102	21.4	9.30	0.002

Parental knowledge of caries and caries prevention is shown in Table 3. Parental opinion about their child's teeth was rated as good by 81.5%. Parents of the two age groups differed significantly in their knowledge about missing school days due to toothache (x^2^ = 21.6, *p* < 0.001), reason for visiting the dentist (x^2^ = 59.5, *p* < 0.001), and fluoride sources (x^2^ = 6.0, *p* = 0.048). Parental knowledge about caries and caries prevention regarding effect of dental caries and periodontal problems on general health and fluoride effect on teeth was significantly better in university educated parents compared to secondary school educated parents in the univariate and multivariate analysis. Age was not a significant factor with regards to any of the variables in Table 3.

**Table 3:** Parental knowledge of caries and caries prevention.

**Variable**	** <6 years old (*****N*** **=** **166)**		**6–12 years old (*****N*** **=** **310)**		**Total (*****N*** **=** **476)**		**Chi-square value**	***P*-value**
	**n**	**%**	**n**	**%**	**N**	**%**		
**Opinion about children's teeth status**							4.6	0.031
Good	144	86.7	244	78.7	388	81.5		
Poor	22	13.3	66	21.3	88	18.5		
**Opinion about the reason for dental decay**							2.5	0.417
Sugar consumption	42	25.3	83	26.8	125	26.3		
Poor oral hygiene	12	7.2	36	11.6	48	10.1		
Both	101	60.8	170	54.8	271	56.9		
Don't know	11	6.6	21	6.8	32	6.7		
**General health is affected by dental caries and periodontal disease**	127	76.5	238	76.8	365	76.7	0.001	0.968
**Missing school days due to toothache**	26	15.7	113	36.5	139	29.2	21.6	<0.001
**Fluoride sources**							6.0	0.048
Tooth paste	77	46.4	180	58.1	257	54.0		
Drinking water	42	25.3	58	18.7	100	21.0		
Don't know	47	28.3	72	23.2	119	25.0		
**Fluoride effect on teeth**							0.5	0.758
Whitening teeth	28	16.9	57	18.4	85	17.9		
Strength and caries resistance	108	65.1	191	61.6	299	62.8		
Don't know	30	18.1	62	20.0	92	19.3		
**Reason for visiting the dentist**							59.5	<0.001
Dental pain	73	44.0	220	71.0	293	61.6		
Check up	15	9.0	45	14.5	60	12.6		
Never been there	78	47.0	45	14.5	123	25.8		
**Knowledge about pediatric dentistry specialty**							2.29	0.318
Yes, and treat there	71	42.8	147	47.4	218	45.8		
Yes, but treat with general practitioner	58	34.9	111	35.8	169	35.5		
Don't know	37	22.3	52	16.8	89	18.7		

Parental knowledge and acceptance of different treatment options of the primary teeth by parents is shown in Table 4. From the provided treatment option list, scaling and extraction were the highest known and accepted treatment by the parents (35.5 and 30.1%, respectively), while treatment under nitrous oxide/ oxygen sedation was the least (3.6%). Comparisons between parental knowledge and acceptance of treatment options were not significant for both children age groups (<6, 6–12) and gender. There was no significant difference in knowledge and acceptance of all the treatment options among the participants according to parental educational level except for composite restorations, fluoride gel application and pulp therapy, where university educated parents knew and accepted those two treatment options more (*P* = 0.03, 0.02, and 0.03, respectively). With regards to age, those who were above 40 years had significantly more knowledge and acceptance of amalgam restorations than those between 20 and 40 years (*P* = 0.04). The findings of the multivariate analysis were consistent with the findings of the univariate analysis.

**Table 4:** Parental knowledge and acceptance of different treatment options in primary teeth.

**Treatment option**	**Knowledge**	**Acceptance**	**Knowledge and acceptance**
Scaling	53.6%	48.8%	35.5%^**^
Fissure Sealant	9%	13.9%	5.4%^*^
Fluoride Gel Application	27.1%	30.7%	18.7%
Composite Restoration	36.1%	42.2%	23.5%
Amalgam Restoration	28.3%	27.7%	17.5%
Pulp therapy	13.9%	12%	7.2%^*^
Stainless steel Crown	11.4%	14.5%	6%^*^
Extraction	48.8%	41.6%	30.1%^**^
Space Maintainer	26.5%	31.3%	18.7%
Nitrous oxide Sedation	6%	10.2%	3.6%^*^
General Anesthesia	15.7%	24.7%	8.4%
Dental Radiographs	34.9%	43.4%	27.1%

* *Treatments less known and accepted by the parents*.

** *Treatments most known and accepted by the parents*.

Responses of parents to the clinical scenarios are indicated in the figure legends of Figures 1, 2. Regarding the importance of dental treatment for the primary dentition as compared to permanent teeth, 55% of the parents stated that primary teeth will be replaced and no need to do any dental treatment. As to whether parents had enough information about oral health of their children, 91.6% of parents were interested in receiving more information about the primary dentition.

The number of decayed, filled and missing teeth were summed together to give the DMFT score for the permanent dentition and the dmft score for the primary dentition (Table 5). The mean dmft and DMFT for the children was 4.64 (SD 3.95) and 1.08 (SD 1.47), respectively. Only 58 (12.2%) of children had dmft = 0, and 32 (6.7%) children had DMFT = 0. The modified O'leary plaque index mean was 0.88 (SD 0.20) with a maximum score of 1 in 66.2% children and a minimum score of 0 in 0.2% which was in one child. Moreover, the modified O'leary gingival index mean was 0.16 (SD 0.23) with a maximum score of 1 in 2.3% and a minimum score of 0 in 50.8% of children. Of the 81.5% parents who reported that their children's dental status was good, only 21.6 and 57.2% had dmft and DMFT = 0, while 78.4 and 42.8% had an average dmft/DMFT score of 5.34/2.32. However, the PI and GI = 0 was present in 11.9 and 74.2%.

**Table 5:** Average DMFT/dmft table for children in the sample by age (years), total sample *n* = 476 (<6 years old = 166, 6–12 years old = 310), % are based on the total sample size.

**Age of child**		**Average DMFT/dmft**	**N (%)**	**Diagnosis**
<6 years old(*n* = 166)	2–3 years	Average dmft = 0	18 (3.8)	Caries free
	40 (8.4%)	Average dmft = 1.6	11 (2.3)	Dental caries (ECC)^*^
		Average dmft ≥ age +1	11 (2.3)	S-ECC^**^
	4–5 years	Average dmft = 0	40 (8.4)	Caries free
	126 (26.5)	Average dmft = 2.9	35 (7.4)	Dental caries (ECC)^*^
		Average dmft ≥ age +1	51 (10.7)	S-ECC^**^
6–12 years old	6–12 years	Average DMFT/dmft = 0	32 (6.7)	Caries free
	310 (65.1)	Average DMFT/dmft = 5.5	278 (58.4)	Dental caries
**Total**		**476**

* *ECC: Early Childhood Caries defined as presence of 1 or more decayed (non-cavitated or cavitated lesions), missing (due to caries), or filled tooth surfaces in any primary tooth in a child 71 month of age or younger (13)*.

** *S-ECC: Any sign of smooth-surface caries in children younger than 3 years of age. From age 3 through 5, 1 or more cavitated, missing (due to caries), or filled smooth surfaces in primary maxillary anterior teeth or a decayed, missing, or filled score of ≥4 (age 3), ≥5 (age 4), ≥6 (age 5) surfaces (13)*.

## Discussion

The preservation of healthy teeth is one of the key health issues in childhood and parents play an important role in their children's oral health. Parental education played a significant role in knowledge and practices toward children's oral hygiene at home with regards to having a toothbrush, brushing frequency and supervision during brushing. A high percentage of children in the sample were reported to brush their teeth at least once to twice daily, similar results were found in other studies (14–16), and the brushing frequency was more when the parents were university educated, similar to that reported by Rajab (17). More than half of the total sample of parents guided and supervised their children while brushing their teeth (Table 2) and this was similar to other's findings (18). The use of additional measures was low in our sample, similar to other studies (16, 19), and this could be attributed to the lack of knowledge of benefits of these auxiliary methods (15, 16, 19, 20). Studies in Jordan on early childhood caries revealed several risk factors associated with caries in children. Some of these factors included; infant feeding habits, oral hygiene practices and socioeconomic status (17, 21, 22). Regarding socioeconomic status there were conflicting results about its actual impact on ECC in Jordan (22–25). Yet, no indices were used to measure oral hygiene and plaque scores and their association with ECC in these studies (26).

In relation to parental knowledge about caries and caries prevention, most of the parents in both age groups recognized the sources of fluoride and its effect on teeth and many of the parents reported that poor oral hygiene and high sugar consumption were the causes for dental decay and this is consistent with other studies (16, 17, 20), and indicates good knowledge about causes of teeth decay. Again, parental education was significantly related to knowledge about effect of dental caries and periodontal problems on general health and fluoride effect on teeth. A high proportion of the subjects reported that children's bad oral health could affect the general health which implies that their children's oral health should be in a good status, however, this wasn't the case since dmft/DMFT was 4.64/1.08. Similar to previous studies (16, 18–20) parents reported that pain was the main reason for visiting the dental office instead of regular check-up.

The lack of knowledge of parents about different treatment options for primary teeth, especially treatment under general anesthesia or nitrous oxide/oxygen sedation that are used in special situations for pediatric patients could be attributed to fear and low dental awareness (5, 6). Additionally, knowledge regarding pulp therapy and stainless steel crowns for primary teeth was found to be very low, and this explains the low parental acceptance of the treatment with these two options and the impact of parental education which was significant compared to those who have less education. Preventive treatment options that can be provided for children such as fissure sealants were neither known by parents nor accepted opposite to what was found in other countries such as in Australia (27), and this could partially explain the high dmft/DMFT and plaque index in the children and the highest knowledge and acceptance for scaling and extraction. Parental education was significantly important in those who recognized fluoride gel application and accepted it as a treatment. Regarding dental filling materials, composite restorations were significantly more known and accepted by university educated parents and those in the 20–40 age group more than amalgam restorations, which indicated that aesthetic was more of a concern than other factors that influence the restoration material selection in this group of participants (28). The need for more knowledge about primary dentition preservation and treatment was in agreement with other studies (29, 30), indicating that more attention should be given for dental educational programs for parents and children.

In the two given scenarios, the majority of parents were happy to leave the decision about the treatment to the dentist and parental educational level or age did not play a significant role in their preferences for treatment. The reliance of parents on dentist for decision on the choice of their children's dental treatment suggested the need for dental health education to both parents and children on dental treatment (4, 6, 7). Notably, 91.6% of parents were interested in receiving more information about the primary dentition. In the asymptomatic carious primary tooth scenario, studies showed that some parents refused any treatment to be provided for a primary tooth as it will be replaced by a permanent successor (4, 6, 20, 29). In the second scenario were the child had a carious primary tooth with toothache requiring pulp therapy and stainless steel crown, some parents decided that the best treatment to be provided for this primary tooth was the extraction to relieve the pain as found by other studies (6, 8). Some parents in other studies reported that primary teeth were important and should be preserved (18, 31).

The poor status of the children's teeth in terms of mean dmft/DMFT scores and plaque index didn't correlate with parental assessment of their child's oral health. Of parents who reported that their children's dental status was good, 78.4 and 42.8% had an average dmft/DMFT score 5.34/ 2.32. Studies in Jordan showed that the overall prevalence of early childhood caries ranges between 48 and 72% (22–24, 32). The plaque index in most children (66.2%) was 1 which indicates presence of dental plaque when the children were examined; this reflects poor oral hygiene practices and predicts more caries and more gingival problems at a young age (6, 15, 33, 34).

There were some limitations for this study as it relied on a self-constructed questionnaire that lacked content and construct validity and was a single center study that didn't assess different regions in Jordan and other socio-economic factors that could have affected the choices by the parents. In addition, Berkson error could have been caused as the study was carried out on patients admitted to a clinic, and results could be subject to bias report given the nature of the study (questionnaire-based) and selection bias, and factors such as differences in maternal and paternal perception of choices of treatment since any of the parents answered the questionnaire based on who attended with the child. On the other hand, a relatively high number of subjects, compliance between observers, clear evaluation criteria and extensive sociodemographic data were the strengths of the study. In conclusion, parental knowledge and acceptance about dental treatment options for primary dentition was generally low. Parental education and age had an impact on parental knowledge and practices regarding the child's oral hygiene, caries and caries prevention, and some treatment options. There was an overrated parental opinion of their child's teeth status despite the high dmft/DMFT, PI and GI in the children. This reflects the need of more effective communication between dental professionals and parents in addition to public preventive and educational programs in order to educate them how to take care of their child's oral health, the importance of regular dental visits and including parents in the decision of treatment instead of the total reliance on dental professionals which could be a recommendation for future studies.

## Data Availability Statement

All datasets generated for this study are included in the article/supplementary material.

## Ethics Statement

Ethical approval was gained from the IRB, JUST (Institutional Review Board, Jordan University of Science and Technology), grant # 175/2014. In addition, written informed consent was obtained from all parents/caregivers after explanation of study objectives.

## Author Contributions

OA-B conceived the research idea, designed the study, and wrote up the manuscript. HA-K performed data collection and data entry. WI assisted in data collection and YK performed data analysis. All authors agreed on the final manuscript.

### Conflict of Interest

The authors declare that the research was conducted in the absence of any commercial or financial relationships that could be construed as a potential conflict of interest.
